# Lived experiences of human immunodeficiency virus and hypertension in the Eastern Cape, South Africa

**DOI:** 10.4102/phcfm.v12i1.2472

**Published:** 2020-10-27

**Authors:** Lwandile Tokwe, Joanne R. Naidoo

**Affiliations:** 1 Department of Nursing Science, Faculty of Health Sciences, Nelson Mandela University, Port Elizabeth, South Africa

**Keywords:** adults, HIV, hypertension, lived experiences, PHC

## Abstract

**Background:**

Globally, the healthcare system is burdened with the rise in communicable diseases compounded by the comorbidity of non-communicable diseases. South Africa in particular experiences a quadruple burden of diseases, and human immunodeficiency virus (HIV) and hypertension are amongst the burden of diseases reported.

**Aim:**

This article aims to explore and describe the lived experiences of people living with HIV (PLWH) and hypertension in the Eastern Cape, South Africa.

**Setting:**

The study was conducted in the Sakhisizwe sub-district within the Chris Hani health district of the Eastern Cape.

**Methods:**

A qualitative study design using Husserl’s descriptive phenomenology underpinned this study. Purposive sampling method was used to select participants. Information was gathered using semi-structured interviews from nine participants who met the inclusion criteria. The interviews were recorded on an audiotape and conducted in isiXhosa, and these were verified through back and forward translation to English. The transcribed interviews were coded manually, and underpinned by Giorgi’s phenomenological data analysis steps.

**Results:**

This study yielded four themes that described the journey towards a new normal experienced by participants. These themes were (1) overcoming illness-related stigma, (2) sources of support, (3) self-love: taking ownership of the diseases and (4) creating transforming behaviours and self-care strategies.

**Conclusion:**

This study demonstrated that the central theme that emerged from the lived experiences of participants with HIV and hypertension was a process of finding a new normal for their lives. This process had several enabling and inhibiting conditions that enabled participants to develop self-acceptance and find strategies to transform behaviours to better live with two chronic illnesses.

## Introduction

Approximately 37.9 million people in 2019 were reported to be living with human immunodeficiency virus (HIV) across the world and of that, 23.3 million people were enrolled on highly active antiretroviral therapy (HAART) and 36.2 million were adults.^1,2^ The recent empirical evidence highlights an increase in people living with HIV (PLWH) from 36.9 million people who were reported in 2018. Further to this, the number of PLWH enrolled on HAART also increased from 21.7 million in 2017 to 23.3 million in 2018, suggesting an increase in the number being diagnosed and enrolled on HAART.^1,3^

Coupled with the burden of disease HIV poses to the global healthcare landscape, non-communicable diseases (NCDs) are reported to further place a burden on the healthcare systems and quality of healthcare.^4,5^ Non-communicable diseases are reported to be the leading cause of death in the world and are the main challenge in the low- and middle-income countries. Further to this, in 2016, NCDs were responsible for approximately 71% of the 57 million global deaths.^6^

There is an association between HIV and hypertension because of several risk factors such as age, weight and those related to HAART medication.^7,8,9^ The established link and rise of hypertension amongst PLWH on chronic medication is also reported to be correlated with poor medication compliance.^10,11^ In addition, non-adherence to chronic medication, in particular in NCDs, tuberculosis and HIV, is a problem that is continuing to increase.^12^

As mentioned by van Zoest et al.^13^ hypertension trends in PLWH are increased than in people not infected with HIV, and the predisposing factors leading to this burden include lifestyle factors, genetics, use of HAART and environmental factors. Supporting this, Medina-Thorne et al.^8^ discussed that there was an increased prevalence of hypertension of 31% amongst PLWH in both male and female participants with a median age of 41 years. South Africa is encountering a dual burden of HIV and hypertension.

In South Africa, the incidence of HIV has increased in comparison with the 2018 statistics of 7.52 million PLWH reported.^14^ Recent empirical evidence by Statistics South Africa^15^ highlights that South Africa is the leading country in the world with 7.97 million PLWH reported, thus making 13.5% of the total population. Whilst HIV continues to rise, hypertension is reported to be highly prevalent amongst PLWH in South Africa, thus increasing the burden of living with the comorbidity of HIV and hypertension. Hypertension has been reported to be the cause of death across the world and approximately 1.13 billion people are hypertensive especially in the low- and middle-income countries.^16^ According to the National Department of Health (NDOH),^17^ 46% of women and 44% of men are living with hypertension in South Africa.

Whilst both HIV and hypertension as chronic illnesses are rising, the burden of the comorbidity of HIV and hypertension has been reported in South Africa.^18,19^ Further to this, it is also demonstrated that this comorbidity is especially prevalent amongst adults who are 40 years; this is evidenced by studies conducted in South Africa.^20,21^ Whilst there may be these reports, there is limited research on the burden of living with HIV and hypertension especially in the rural areas of Eastern Cape in South Africa. In view of this, our study sought to explore the lived experiences of PLWH and hypertension in the Eastern Cape province of South Africa. This study also sought to explore the facilitating and inhibitory factors experienced by PLWH and hypertension.

## Research methods and design

### Study design

This was a qualitative study utilising Husserl’s descriptive phenomenology and semi-structured interviews to gather data.

### Setting

This study was conducted in the Sakhisizwe sub-district of the Eastern Cape, South Africa. It is a rural area in nature, with few buildings and limited shops in town; people are involved in subsistence farming. The town is surrounded by the Tsomo River and mountains with trees and has a total population of 63 582.^22^ The sub-district has 13 fixed clinics and one mobile clinic. The primary healthcare (PHC) services offered include comprehensive chronic care, acute care, mother and child healthcare, family planning, mental healthcare and sexually transmitted infection management.

This setting was chosen because of the high prevalence of HIV and hypertension based on the observation of the researcher who worked as a PHC nurse in the same health district that PLWH are also living with hypertension as a comorbid illness. The Sakhisizwe sub-district is one of six sub-districts in the Chris Hani Municipality of the Eastern Cape. All the participants met the inclusion criteria of the study, which was that they should be living with comorbidity of HIV and hypertension for at least 1 year and be 40 years and older.

### Study population, sampling strategy and recruitment

The age range of the participants was between 40 and 59 years, with five participants being females and four being males. The average age of the participants was 49 years.

The participants were invited to take part in the study and recruitment involved explaining the study to the participants with the assistance of the information sheet drafted. Purposive sampling method was used to select the participants living with HIV and hypertension at the Sakhisizwe sub-district and were identified. The participants were invited to participate as they came for their monthly chronic medication visits by explaining the study to them, which included the title, purpose, aim, benefits, risks and confidentiality of information, and asked for their consent.

### Data gathering

The data gathering method used in this study included semi-structured interviews that entailed asking questions and the use of probes by the researcher.^23^ The researcher implemented this by making use of the semi-structured interview schedule that contained the probes that were used to extract meaningful responses from the participants. Data gathering commenced on 01 November 2018, and written consent was obtained from all the participants.

All patients were Xhosa speakers; hence, interviews were held in *isiXhos*a, audiotaped and transcribed verbatim. The interviews were gathered by the primary investigator. Transcripts were coded by using pseudonyms to ensure ethical principles of confidentiality, and anonymity was maintained. Back translation was conducted and verified by a language expert to ensure that the meaning from the translated transcripts was not lost.

## Data analysis

Giorgi’s phenomenological steps of data analysis were used in the analysis of the data.^24^ Field notes were recorded between the interviews and a reflective journal was used to achieve the methodological stance of bracketing.

### Ethical consideration

Ethical clearance was granted from the Nelson Mandela University Human Research Ethics Committee (H18-HEA-NUR-007). Permission was sought from the Eastern Cape Department of Health (EC_201810_002) and the respective health managers from the Chris Hani and Sakhisizwe districts. Data gathering commenced on 01 November 2018, and written consent was obtained from all the participants.

## Results

The results of this study were structured by initially describing the demographic characteristics of the participants. Secondly, the themes and sub-themes were outlined. Representative quotes from the participants are also included to illustrate the findings of this study from all themes and sub-themes.

### Demographic characteristics of the participants

A total of nine participants were interviewed. All participants were from the *AmaXhosa* ethnic group and were all living within the catchment areas of the selected facility within the Sakhisizwe sub-district. In terms of educational status, none of the participants had completed educational training higher than grade 12 and only two participants had completed grade 12. The participants had an average number of two dependents.

In terms of years living with HIV and hypertension separately prior to the comorbidity, the average number of years living with HIV was 9 years and 7 years for hypertension. In the sample of participants who were living with HIV only longer than living with the comorbidity of HIV and hypertension, there was an average of 6 years and in the sample of participants who were living with the comorbidity of HIV and hypertension there was a range of 3–10 years. Table 1 represents the demographic characteristics of the participants.

**TABLE 1:** Participants’ demographic characteristics.

Pseudonym	Age	Gender	Highest level of education	Years of living with HIV and hypertension			Number of dependents
				Years of living with HIV only	Years of living with hypertension only	Years of living with HIV and hypertension	
Bulelwa	44	Female	Grade 11	3	3	3	02
Nolubabalo	48	Female	Grade 12	9	9	9	03
Fumanekile	57	Male	Grade 9	18	4	4	04
Nosigniture	55	Female	Grade 8	7	2	2	02
Sipho	55	Male	Grade 8	5	5	5	02
Zandile	41	Female	Grade 12	7	15	7	02
Siyanda	43	Male	Grade 6	5	2	2	01
Lelethu	46	Female	Grade 10	9	10	9	04
Vuyani	52	Male	Grade 10	15	10	10	02

HIV, human immunodeficiency virus.

The findings of this study revealed four themes and 14 sub-themes on the participants’ descriptions of the lived experiences of living with HIV and hypertension. The themes and sub-themes are outlined in Table 2.

**TABLE 2:** Themes and sub-themes.

Themes	Sub-themes
Overcoming illness-related stigma	Anticipated stigma Internal stigma External stigma
Sources of support	Family support Peer support Health practitioner’s support
Self-love: Taking ownership of the diseases	Self-acceptance Self-motivation
Creating transforming behaviours and self-care strategies	Making a plan Coping with medication Access to treatment Grading of diseases Pill burden Unhealthy addictions

### Theme 1: Overcoming illness-related stigma

The participants expressed that living with HIV and hypertension was plagued with stigma related to their illness, in particular experiencing stigma that was related to the participants’ HIV status. Participants experienced being judged and labelled with the virus by the people in the rural community. Various forms of illness-related stigmas emerged as a theme that participants experienced in various ways, and learning to overcome the stigma and discrimination formed part of the process participants experienced towards developing a new normal life as the lived experience of living with HIV and hypertension in terms of their disease management. Participants experienced stigma as manifesting in three forms, namely anticipated stigma, internal stigma and external stigma. Participants identified that overcoming the various forms of stigma and discrimination was a process that varied for each participant and was dependent on their acceptance of their HIV or hypertension diagnosis:

‘I was scared that they will gossip and say things you see. I don’t know this condition, it is not lucky.’ (T1, L 641–642)

#### Sub-theme 1: Anticipated stigma

Anticipated stigma was experienced in terms of participants expecting a negative behaviour or outcome from their family or significant others in the light of living with HIV. Participants experienced anxiety of disclosing their HIV status because of the fear of the reaction from their family or significant others:

‘I was afraid because I didn’t know if they were going to accept.’ (T6, L 122–123)

#### Sub-theme 2: Internal stigma

Evident from the data analysis, the participants verbalised internal stigma that occurred within them and was directed by self. This type of stigma made them to experience feelings of self-blame with regard to the lifestyle and life choices that they had made and adopted before acquiring the illnesses:

‘I blamed myself because I knew the way I was carrying myself wrong you know is where I had HIV you see. Yes for not using a condom when sleeping with people you see. So those were the mistakes of alcohol even so you see. So that is why I’m saying I was not scared when it was said I have HIV because I knew that I was playing careless you know.’ (T7, L 463–470)

The participants experienced feelings of regret towards a new normal of living with HIV and hypertension and they started to stigmatise themselves to a point where they started to regret everything and how they had behaved in the past:

‘I regretted my behaviour double; I regretted myself double.’ (T5, L 560)

#### Sub-theme 3: External stigma

Based on the findings of the study, the participants verbalised fear of being stigmatised by the people in the rural community because of their diagnoses. The participants experienced extreme anxiety of being stigmatised by members of the community and being known that they are living with HIV. In addition, some participants towards a new normal of living with HIV experienced that when they were going in places where there were people, they would remove their medications from their containers to minimise the noise caused by the pills that attracts attention of people in their community and results in stigmatisation:

‘Sometimes I put it inside the container and wrap it with a toilet paper so that it stops the movement that makes the noise.’ (T9, L 734–735)

Furthermore, some of the participants verbalised that they feared how the negative remarks from the people in the community would make them feel when they discovered that they were living with HIV; hence, they end up hiding their diagnoses. The following statements explain this further:

‘I was scared that they will go on spreading to everyone that this one is sick, this and that.’ (T2, L 1078–1079)

In addition, some participants towards developing a new normal of living with HIV experienced fear of being identified by their illnesses and losing their worth in the community:

‘Maybe every person when looking at me will look and say this person is living with the virus.’ (T1, L 159–160)

### Theme 2: Sources of support

Based on the findings of the study, the participants experienced that family support played a major role in helping them towards developing a new normal such that their support enabled the participants to accept their diagnoses, to accept themselves and to adhere to their medication. The following excerpts reflect this further:

‘Yes my family, they said that if you take my pills, that is what we are saying nothing else, if you are taking your treatment the right way, you will be alright.’ (T2, L 250–252)

‘Ey my brother I don’t want to lie, I received support from my family there is nothing that is beyond family.’ (T6, L 455–456)

The participants expressed that because they lived in a rural community where stigma is a challenge, their families and loved ones were their source of strength who encouraged them and showed them love and advised them to continue to use their medication. The participants experienced support from their parents, who accepted them when they disclosed to them, and from their siblings:

‘She’d encourage me to cook on time so that I can eat your pills (Referring to her mother).’ (T2, L 380)

In addition, some of the participants verbalised that for them, their significant others, who include their wives and children, were their sources of support in living with two illnesses. Some participants verbalised that their partners played a role in supporting them and enabled them to cope with living with two illnesses. The quote reflects this further:

‘It’s my wife. She will say father of my child, take your treatment. After eating your porridge in the morning and late take your treatment and not forget it.’ (T5, L 444–445)

#### Sub-theme 1: Family support

The participants reported that because they live in a rural community where stigma is still a challenge, their families and loved ones were their source of strength who encouraged them and showed them love and advised them to continue to use their medication. The participants experienced support from their parents, who accepted them when they disclosed to them, and from their siblings:

‘I told my mother first. yes, then I told my husband.’ (T6, L 117–119)

In addition, some of the participants verbalised that for them, their significant others, who include their wives and children, were their sources of support in living with two illnesses:

‘He supports me and I support him or when he has arrived and I see that he is busy and its time to take his pill, I bring it to him.’ (T8, L 431–432)

#### Sub-theme 2: Peer support

Evident from the data analysis, the participants verbalised that although their families and significant others supported them, support of their peers played a crucial role in enabling them to accept themselves and comply with the treatment of the two illnesses. In addition, the participants expressed that for them even when they were diagnosed, their peers were amongst the people who initially supported them:

‘No I told my friends whom I chat with.’ (T2, L 260)

Furthermore, the participants experienced the peer support through other patients whom they knew and just like them live with the chronic illnesses, and they received support from each other. Although the participants expressed that they did not have support groups, they verbalised that they relied on each other for support so that they can cope living with the illnesses:

‘They just said my sister we are living with pills since we were started in years ago because they started before me. We are still taking our pills they said and there is nothing as you can see us we are still walking and alive.’ (T2, L 581–584)

Moreover, the participants expressed that they experienced support of their peers through their meaningful conversations when they are in the clinic for their monthly medication:

‘Others as we are sitting at the clinic, others the person will say no if you eat treatment in the right way, there is nothing.’ (T1, L 210–211)

Some of the participants expressed that they even reminded each other to continue taking their medication for their illness rather than being defeated by medication:

‘There are a lot that that I chat with, we even remind each other not be defeated by one pill, the one we take at eight.’ (T4, L 694)

#### Sub-theme 3: Health practitioner’s support

It emerged from the data that although the participants received support from their families and peers, the professional nurses in the clinic were also amongst the people whom they confided in and who provided support and encouragement to them and gave them hope towards developing a new normal of living with HIV and hypertension. The participants verbalised that being in the rural area, they regarded the nurses as the influential people who have played a part in them coping with the two illnesses. In addition, the participants verbalised that for them, the nurses were also their source of support who encouraged them to accept themselves and who helped them to cope with the two illnesses through the education and counselling they provide in the clinic:

‘To me it is treatment there is nothing else that is except that, and the On-going counselling by the nurses.’ (T2, L 246–247)

Furthermore, the participants expressed that the professional nurses in the clinic they attend were always welcoming and were the people who were always eager to assist them in coping with the illnesses and advising them:

‘They sat down with me first and it was explained to that I must know that if we check and find that you have this and this, you mustn’t be shocked you see. You should accept and I said no problem check me everything that you want to check you see.’ (T7, L188–191)

Moreover, based on the findings, the participants verbalised that the professional nurses in the clinic were their source of information and support, and they were always displaying a positive attitude that made it possible for them to confide in them:

‘The way nurses are treating us is well because would find out that the nurse compliments you and say you are beautiful, you are clean and your skin.’

‘Those encouraging words. Your skin is well. You are taking good care of yourself.’ (T1, 526–569)

The participants experienced support from the nurses through the way they communicated with them and through the way they always went an extra mile to make sure that they continued to take their medication both for HIV and hypertension. The participants verbalised that although there was still stigma, the neutral place was the clinic with the nurses who were always consistent in the care they provided and their support:

‘When I come here at the clinic, I’m helped by my nurses Whom I take treatment to then I gain strength.’ (T5, L 530–532)

Evident from the data, the participants expressed that the support of nurses was what enabled them to accept themselves and their diagnosis, thus facilitating the process of living with two chronic illnesses. The participants explained that the nurses were what enabled them towards a new normal.

### Theme 3: Self-love: Taking ownership of the diseases

The third theme that emerged from the data analysis of taking ownership and loving themselves was what enabled the participants to cope with living with HIV and hypertension. The participants experienced self-acceptance and motivation as a means of coping towards developing a new normal with HIV and hypertension. This is demonstrated by the development of self-encouragement in the management of the two illnesses:

‘No things that helped me to cope with this treatment is one knowing that if I don’t take my treatment, the condition will be worse so I need to careful and go and take treatment so that, instead of the condition worsening, it will be better.’ (T3, L 286–289)

#### Sub-theme 1: Self-acceptance

Evident from the data, self-acceptance for the participants meant that for them to be able to live with the two illnesses they had to first accept themselves as the people who were living with the two illnesses:

‘I accepted that then. I felt strong because knowing that it is a condition that is present in people and it is not only in me only.’ (T3, L 85–88).

#### Sub-theme 2: Self-motivation

The participants described that self-motivation was what enabled them to cope and take their medication for the two illnesses. Participants expressed that they experienced this by encouraging themselves to continue to adopt appropriate behaviour, adhering to medication and becoming responsible towards their management of the two illnesses:

‘I told myself that if I don’t take my treatment it is the end of my life.’ (T8, L 330–331).

### Theme 4: Creating transforming behaviours and self-care strategies

Based on the data analysis, the fourth theme that emerged was making healthy life choices: HIV was better than hypertension. The participants expressed that in order to continue to live with the two chronic illnesses, they had to start taking good care of themselves towards developing a new normal of HIV and hypertension. This meant that the participants had to change the way they were eating and following instructions given:

‘When you have these conditions, you don’t drink alcohol, you don’t smoke cigarette, yes and you need to be a person who love drinking water the food must not have excess of fat, and a lot of salt.’ (T3, L 423–435)

#### Sub-theme 1: Making a plan

It emerged from the data that in order for the participants to take care of themselves, there was a need to develop a plan that would facilitate how they managed their two chronic illnesses well. The participants expressed that the plan included preparing their meals prior to the time of taking the medication so that they could be able to take their medication at their specified times, which is in the morning and in the afternoon in order to accommodate their anti-hypertensive drugs and anti-viral drugs. In addition, the participants experienced that to manage these two illnesses required them to adopt a healthy lifestyle, which meant that the diet that they consumed should be the one that has the necessary nutrients needed by the body:

‘I eat healthy food; fruits and veg, meat and so on.’ (T2, L 706–707)

#### Sub-theme 2: Coping with medication

The participants expressed that achieving self-care meant that one had to take one’s medication everyday as the conditions were chronic and required daily intake of the medication. This meant that the participants needed to practise good adherence to medications for both HIV and hypertension:

‘I leave home at 07h00 and by 06h30 maybe I would’ve already been up and by 06h55 I swallow my pill before going out.’ (T2, L 611–13)

#### Sub-theme 3: Access to treatment

Based on the data analysis, the participants expressed that towards a new normal, one must access care that was not always within their reach. The participants verbalised that being in a rural area and having one clinic meant that they had to access medication at that one clinic. In addition, the participants experienced that achieving self-care for them meant that they had to travel a long distance to receive medication for their chronic illnesses as the clinic was far:

‘The clinic is far when you think that you woke up early to go the clinic, you are afraid of hiking and arrive there that there are a lot of people and you are the last one.’ (T6, L 709–711)

#### Sub-theme 4: Grading of diseases

Evident from the data, the participants experienced grading of their illnesses. The grading was marked by the comparison that the participants made regarding their illnesses in terms of which one was life threatening and serious as compared to the other. Some participants expressed that hypertension for them was more serious than HIV therefore requiring more care:

‘But I saw that by looking, HIV is better than pressure that I have because with pressure, it does go down or anything, I try to eat things I am told to eat but it is always high.’ (T1, L 143–146)

#### Sub-theme 5: Pill burden

Evident from the data, the participants expressed the transition in the lifestyle through the use of the medication for the two chronic illnesses. The participants verbalised that some of them experienced treatment fatigue because of the number of pills they had to take a day for HIV and hypertension:

‘I started the time the pills were more, they were still more than one it was four different kinds. I used to take others at the same time others I would separate them and take them at different time.’ (T5, L 312–315)

#### Sub-theme 6: Unhealthy addictions

The participants verbalised the experience of change in the lifestyle of adopting unhealthy addictions as a way to coping with living with HIV and hypertension. The change was verbalised as developing an addiction to smoking and heavy alcohol intake that they experienced as a barrier towards the lifestyle:

‘I do drink alcohol but I don’t smoke. No I do drink alcohol. Yes I sometimes have that problem when I drink alcohol day before going to clinic you know and my blood pressure becomes high you know. So this is where I suspect that I am using alcohol heavily sometimes I’ve seen that my pressure becomes high when I have used alcohol heavily.’ (T7, L 516–518)

## Discussion

Our study provides an insight into the lived experiences of PLWH and hypertension in the Eastern Cape. The findings relate to the setting of the study not the whole of the Eastern Cape. The findings of this study revealed that the participants experienced three types of stigma, which included internal stigma, external stigma and anticipated stigma associated with the HIV diagnosis. In addition, the findings also revealed that the patients experienced support from different influential figures who enabled them to cope with living with the comorbidity of HIV and hypertension.

The findings of the study further revealed that self-love in the form of self-acceptance of the illnesses and self-motivation enabled the participants to cope with living with the comorbidity. Moreover, the findings of this study also revealed that living with HIV and hypertension required participants to adopt self-care strategies that include lifestyle modification and adherence to treatment for both illnesses. Lastly, the findings also demonstrated that although there were facilitating factors experienced, inhibitory conditions, which included barriers in accessing treatment, and unhealthy addictions were experienced by other participants.

The trends of stigmatisation and discrimination amongst PLWH have been noted and reported in an African study by Hall et al.^25^ A study by Hill et al.,^26^ which explored the role of the social support amongst PLWH, found that family support was reported to play a huge role in caring for HIV. A study by Iwelunmor et al.^27^ highlighted how some of the participants described peer support as important in the management of a chronic illness of HIV and for sharing feelings.

According to a study by Earnshaw et al.,^28^ the participants also revealed that acceptance of the illness and the use of medication is what enabled them to cope with the chronic illnesses. Moreover, in a study by Angwenyi et al.^29^ it was reported that in making a plan for management of HIV and hypertension as a chronic illness, the participants who were involved in the study had a high awareness regarding food restrictions, especially those living with hypertension, and PLWH were reported to consume a nutritious diet and following the diet plan as per their health provider’s instructions. The findings of this study were consistent with studies that have been conducted. In our knowledge, this is the first study to explore the lived experiences of PLWH and hypertension in the Eastern Cape, South Africa.

The strengths of the study were facilitated by the research methodology and research approach that underpinned the study. In addition, the strengths lay in the use of an exploratory methodology such as that of phenomenology that enabled each participant to share their unique experiences of living with HIV and hypertension.

This study only focused on a smaller sample of nine participants, in one sub-district and in one district. Therefore, the study findings reflected the lived experiences of the patients of the selected setting. In addition, the study was conducted only in one province so it looked only at the Eastern Cape rather than the all South African provinces where patients are living with NCDs and communicable diseases. Furthermore, the study was conducted only to participants who were homogeneous in terms of ethnic group. This meant that the researcher could not generalise the study findings to other areas outside the Eastern Cape and different homogeneous ethnicity.

This study recommends that the research on the lived experiences of living with HIV and hypertension should be conducted on a wider sample and in different provinces so as to get the essence of the experiences of living with two chronic conditions on a wider scale.

## Conclusion

The participants in this study demonstrated that the central theme of their lived experiences of living with HIV and hypertension was the process of finding a new normal of living in the rural areas of the Eastern Cape. This process had facilitating experiences that include self-love, self-care practices and support that enabled the participants to cope with a new normal of living with HIV and hypertension. However, there were inhibitory experiences that include stigma and access to treatment.
